# Correction to “Effectiveness and Accuracy of a Patient‐Specific Instrumentation System for Total Hip Arthroplasty”

**DOI:** 10.1111/os.13734

**Published:** 2023-04-04

Zhang, T. , Jia, Z. , Han, W. , Wang, J. , Li, J. , Gong, M. and Jiang, X. (2023), Effectiveness and accuracy of a patient‐specific instrumentation system for Total hip arthroplasty. Orthop Surg, 15: 878–887. 10.1111/os.13665 36636925PMC9977596

The funding information was missing in the published version. Thus, it should be read as:

## FUNDING INFORMATION

This study was funded by Beijing Natural Science Foundation (L202028) and (L212009).

We apologize for this error.

